# Effect of ultrasound-guided genicular nerve neurolysis versus sham procedure on pain in patients with knee osteoarthritis: a randomized clinical trial

**DOI:** 10.1093/pm/pnaf081

**Published:** 2025-06-24

**Authors:** Malgorzata Reysner, Tomasz Reysner, Grzegorz Kowalski, Aleksander Mularski, Przemyslaw Daroszewski, Katarzyna Wieczorowska-Tobis

**Affiliations:** Department of Palliative Medicine, Poznan University of Medical Sciences, 61-701 Poznań, Poland; Department of Palliative Medicine, Poznan University of Medical Sciences, 61-701 Poznań, Poland; Department of Palliative Medicine, Poznan University of Medical Sciences, 61-701 Poznań, Poland; Department of Forensic Medicine, Institute of Medical Sciences Collegium Medicum, University of Zielona Góra, 65-417 Zielona Góra, Poland; Department of Organization and Management in Health Care, Poznan University of Medical Sciences, 61-701 Poznań, Poland; Department of Palliative Medicine, Poznan University of Medical Sciences, 61-701 Poznań, Poland

**Keywords:** patients, neurolysis, pain, osteoarthritis, knee, elderly, genicular nerves

## Abstract

**Importance:**

Knee osteoarthritis (OA) is a prevalent and disabling condition, especially in older adults. For patients who are not candidates for total knee arthroplasty (TKA) due to comorbidities or limited access to care, minimally invasive pain-relief options are critically needed.

**Objective:**

To assess the efficacy and safety of ultrasound-guided chemical neurolysis using 95% ethanol targeting four genicular nerves in patients with symptomatic knee OA who have not responded to conservative management.

**Design:**

Double-blind, randomized, sham-controlled clinical trial.

**Participants:**

A total of 100 adults with symptomatic knee OA, confirmed radiographically and unresponsive to conservative treatment, were enrolled and randomized.

**Interventions:**

Patients were randomized to receive ultrasound-guided chemical neurolysis of the superomedial, superolateral, recurrent tibial, and inferomedial genicular nerves with 95% ethanol (treatment group) or a sham procedure (control group).

**Main Outcomes and Measures:**

The primary outcome was pain intensity measured by the Numerical Rating Scale (NRS) at 7 days, 30 days, 3 months, and 6 months post-procedure. Secondary outcomes included opioid consumption and health-related quality of life measured by the EQ-5D-5L questionnaire. Safety outcomes focused on the occurrence of neurological complications. Analyses were conducted using an intention-to-treat approach with appropriate handling of missing data.

**Results:**

Patients in the ethanol neurolysis group experienced significantly greater reductions in NRS scores at all follow-up points compared to the sham group (*P* < .0001). Opioid consumption was also significantly reduced in the neurolysis group throughout the 6-month period (*P* < .0001). Quality of life, as assessed by EQ-5D-5L, improved significantly in the treatment group (*P* < .0001). No neurological deficits or serious adverse events were reported in either group.

**Conclusions and Relevance:**

Ultrasound-guided chemical neurolysis of the SMGN, SLGN, RTGN, and IMGN with 95% ethanol is a safe and effective minimally invasive treatment for patients with refractory knee OA pain. It significantly reduces pain and opioid use while improving quality of life, making it a valuable therapeutic option for individuals ineligible for surgical intervention.

## Teaser

This double-blind, randomized controlled trial evaluates the efficacy and safety of ultrasound-guided genicular nerve neurolysis using 95% ethanol in elderly patients with knee osteoarthritis. The intervention significantly reduced pain intensity and opioid use and improved quality of life compared to a sham procedure. No neurological complications were observed. Findings support this minimally invasive technique as a safe and cost-effective alternative for patients ineligible for surgery.

## Introduction

Knee osteoarthritis (OA) is a leading cause of chronic pain and disability, particularly in older patients.^1^ Characterized by progressive cartilage degeneration, joint stiffness, and pain, OA significantly impairs mobility and quality of life.^2^ Current treatment options for knee OA primarily focus on symptom relief, ranging from nonsteroidal anti-inflammatory drugs (NSAIDs) and physical therapy to intra-articular injections and, in advanced cases, joint replacement surgery.^3^ However, many patients, especially patients with symptomatic knee osteoarthritis with comorbidities, may not achieve satisfactory pain control with oral analgesics, leading to a substantial unmet clinical need for alternative therapeutic approaches.^4^

Total knee arthroplasty (TKA) is often considered the definitive treatment for advanced knee OA.^5^^,^^6^ However, TKA, particularly in patients with multiple comorbidities, may be contraindicated due to the increased risk of surgical complications and prolonged recovery.^7^^,^^8^ Moreover, dissatisfaction following TKA remains a critical issue, with approximately 20% of patients reporting persistent pain or limited functional improvement, even after successful implantation.^9^^,^^10^ Additionally, in many less-developed countries, limited medical resources make TKA inaccessible to most patients who require it. This creates a need for cost-effective, minimally invasive alternatives to manage pain in these populations.

Chemical neurolysis, such as ethanol, offers a potentially safe and affordable option for pain treatment in patients with symptomatic osteoarthritis.^11^ While glycerin and phenol have historically been used for chemical neurolysis, ethanol provides several advantages, including rapid onset of action, ease of handling, and lower viscosity, allowing precise tissue infiltration. Additionally, ethanol is widely available, cost-effective, and has a well-documented safety profile in peripheral nerve applications.^12^^,^^13^ Unlike radiofrequency or cryoablation, which require specialized and expensive equipment, chemical neurolysis does not necessitate such resources, making the procedure inexpensive and more widely available. For instance, the estimated cost of radiofrequency ablation (RFA) ranges between $3000 and $5000 per session, whereas ethanol ablation—including the drug and required equipment—can be performed for approximately $100-$200. Chemical neurolytics, such as ethanol, relieve pain by degenerating afferent sensory fibers, effectively disrupting nociceptive transmission from the knee joint.^14^ Ethanol can be easily employed to ablate genicular nerves, providing a feasible alternative for pain management in resource-limited settings.

One promising modality for pain management in knee OA is using genicular nerve blocks or neurolysis.^14^ The genicular nerves, which provide sensory innervation to the knee joint, have emerged as viable targets for pain relief in OA.^15^ Chemical neurolysis using agents such as ethanol aims to disrupt the transmission of nociceptive signals from the knee joint, thereby reducing pain.^16^ Ethanol-induced neurolysis has been shown to produce Wallerian degeneration of sensory nerve fibers, resulting in sustained pain relief.^17^ Prior studies have demonstrated that neurolysis can provide significant pain relief in patients with refractory knee OA. However, the efficacy and safety of different techniques and agents remain subjects of ongoing investigation.^14^

This study aims to evaluate the efficacy and safety of ultrasound-guided chemical ablation of genicular nerves with 95% ethanol in patients with symptomatic knee OA. We hypothesize that this technique will provide significant pain relief, reduce opioid consumption, and improve quality of life compared to sham procedures. All patients in the study had previously attempted standard oral analgesics—including NSAIDs, paracetamol, and co-analgesics—without sufficient symptom relief. Concurrent pharmacologic therapies were continued during the trial when clinically indicated. This study aims to fill a critical gap in the literature by offering robust data on the clinical utility of this approach, especially in patients who have not responded adequately to oral analgesics.

## Methods

### Trial design

Before recruitment, this double-blind, single-center, prospective, randomized controlled trial (NCT06087601) was registered at ClinicalTrials.gov on October 10, 2023. Ethics approval was obtained on March 9, 2023. The study was conducted following the Declaration of Helsinki and was regulated by standards approved by the Consolidated Standards of Reporting Trials (CONSORT) statement. Before enrollment, written informed consent was obtained from all patients' caregivers. The recruitment period extended from October 17, 2023, to January 10, 2025.

### Eligibility criteria

Adult patients of the Pain Treatment Clinic of the Transfiguration of Jesus Clinical Hospital of the Poznan University of Medical Sciences with gonarthrosis who failed to achieve satisfactory pain control (NRS > 3) despite the use of oral analgesics, including NSAIDs, paracetamol, and co-analgesics were approached for participation in the study. Radiographic confirmation using Kellgren–Lawrence (KL) grading was required for eligibility. Only patients with KL grades 3 or 4 were included, determined through standardized anterior-posterior and lateral knee radiographs during the screening process. Exclusion criteria included (1) suspected or diagnosed opioid dependence syndrome, (2) active cancer disease, and (3) dementia. Additional exclusion criteria included (4) previous surgical intervention on the affected knee, such as arthroscopy or osteotomy, and (5) prior TKA.

### Randomization

The randomization process was carefully designed to ensure an unbiased allocation of participants into the study groups while maintaining balance between the 2 arms. A computer-generated randomization list was created using the nQuery Advisor program (Statistical Solutions, Boston, MA, USA), which assigned participants in a 1:1 ratio to either the ultrasound-guided 95% ethanol neurolysis group or the sham neurolysis group. The randomization sequence was based on a permuted block design with randomly selected block sizes to prevent predictability in allocation.

To maintain allocation concealment and prevent selection bias, an independent researcher who was not involved in patient recruitment, intervention, or outcome assessment generated the randomization sequence in advance. The sequence was placed in sequentially numbered, opaque, and sealed envelopes, each containing the group assignment corresponding to a specific randomization number. When a patient met the eligibility criteria and provided informed consent, the study coordinator opened the next sequentially numbered envelope in the presence of a separate investigator responsible for performing the intervention. The study coordinator had no prior knowledge of the group assignment, ensuring an unbiased allocation process.

### Blinding

Blinding was strictly maintained throughout the study to uphold the double-blind design. Patients remained unaware of their assigned intervention, as both neurolysis and sham procedures were performed using the same ultrasound-guided technique. The outcome assessors, including physicians and researchers evaluating primary and secondary endpoints, were blinded to the treatment allocation. The interventionist performing the procedure was the only individual aware of the assignment but had no involvement in data collection or patient follow-up.

The randomization remained concealed until the statistical analysis was completed. Unblinding was only permitted if a severe adverse event or a safety concern necessitated immediate medical intervention. This rigorous randomization ensured the study’s integrity, balance, and validity while preventing potential biases in allocation, treatment administration, and outcome assessment.

### Diagnostic genicular nerve block

Ultrasound-guided diagnostic superomedial genicular nerve (SMGN), superolateral genicular nerve (SLGN), recurrent fibular genicular nerve (RFGN), and inferomedial genicular nerve (IMGN) blocks were performed before procedures. A linear, low-frequency 4-8 MHz sonographic ultrasound probe and a 22-gauge needle (Stimuplex Ultra 360, 90 mm) were used for the procedure. The transducer was placed along the long axis of the femur at the junction of epiphysis and diaphysis. The SMGN, SLGN, RTGN, and IMGN were localized using osseous and muscular landmarks, with the IMGN targeted specifically along its course over the tibial shaft near the medial tibial plateau. The needle was introduced in-plane from anterior to posterior direction. The correct needle position was confirmed by hydro location with 0.5 mL 0.9%NaCl, followed by 1 mL of 2% lignocaine at each site to confirm nerve contact and assess for responsiveness prior to neurolysis. The volume was intentionally higher than the therapeutic injection to ensure adequate coverage during the prognostic phase. Genicular nerves were successfully visualized in all patients.

### 95% Ethanol neurolysis or sham neurolysis

The patients were reassessed 1 week after the diagnostic block. Patients who reported a >50% reduction in knee pain for at least 6 hours were submitted to the neurolytic block. The technique for 95% ethanol neurolysis or sham neurolysis followed the same sonographic and needle guidance protocol used for the prognostic block. Each nerve target was anesthetized with 2% lignocaine. Five minutes later, neurolysis was performed using 0.5 mL of 95% ethanol or 0.5 mL of 0.9% NaCl at each target nerve. The lower injectate volume for neurolysis was deliberately chosen to reduce the risk of off-target spread of ethanol.

After the intervention, patients were advised to resume daily activities within 24 hours while avoiding strenuous physical exertion for 1 week. Oral analgesics such as paracetamol or NSAIDs were permitted on an as-needed basis, but opioids were discouraged unless pain intensity exceeded NRS 4. No physiotherapy referrals were routinely issued, although patients were advised to maintain normal joint mobility through light activity.

### Outcome measures

#### Primary outcome

At all postprocedure time points, during follow-up appointments (7 days, 30 days, 3 months, and 6 months after the procedure), the pain score was assessed using the numeric rating scale (NRS) score (0 meaning no pain and 10 meaning the worst pain imaginable). Patients self-reported NRS values during in-clinic visits using standardized scales.

#### Secondary outcomes

The total opioid consumption, expressed in milligrams of oral morphine per day, and neurological deficits were accessed (7 days, 30 days, 3 months, and 6 months after the procedure) by trained interviewers blinded to allocation. Opioid consumption data were obtained from patient-reported daily medication diaries, which were reviewed and cross-referenced with electronic medical record (EMR) prescriptions to ensure consistency and accuracy. All opioid use was converted to oral morphine milligram equivalents (MME) for standardized reporting.

The Polish version of the EQ-5D-5L questionnaire was also completed at each follow-up visit.^18^ The EQ-5D-5L comprises 5 dimensions: Mobility, self-care, usual activities, pain/discomfort, and anxiety/depression. Each dimension has 5 levels: no problems, slight problems, moderate problems, severe problems, and extreme problems. The patient was asked to indicate their health state by ticking the box next to the most appropriate statement of the 5 dimensions. This decision resulted in a 1-digit number that expressed the level selected for that dimension. The digits for the 5 dimensions were summarized into a number that describes the patient's health state.

Categorical responder rates, including ≥50% NRS reduction and opioid discontinuation rates, were calculated and reported for relevant intervals.

### Sample size calculation

The sample size determination was based on a power analysis for a paired **t-test**, reflecting the primary hypothesis that genicular nerve neurolysis improves pain management in patients with knee osteoarthritis. A retrospective evaluation of unpublished clinical data from the Pain Treatment Clinic provided an estimated mean Numerical Rating Scale (NRS) score of 4 ± 3. To detect a 25% reduction in NRS scores at 7 days post-procedure, with a significance level (α) of 0.05 and a statistical power (1-β) of 0.80, the required sample size was calculated using a 2-sided chi-square (χ^2^) test, yielding a total of 94 participants.

To compensate for potential attrition due to loss to follow-up or unforeseen study discontinuations, an additional 5% dropout rate was incorporated into the calculation. Consequently, the final sample size was adjusted to 100 participants, with 50 patients per study group. This sample size was sufficient to provide statistically robust conclusions regarding the efficacy of ultrasound-guided 95% ethanol neurolysis compared to the sham procedure.

### Statistical analysis

All primary and secondary endpoints were analyzed using an intention-to-treat approach under a superiority framework. Descriptive statistics were reported as mean ± standard deviation (SD) for continuous variables and as frequencies and percentages for categorical variables. The Shapiro–Wilk test was used to assess normality. Between-group comparisons for normally distributed continuous variables were performed using unpaired 2-sample t-tests, while non-normally distributed variables were compared using the Mann–Whitney U test. Categorical variables were assessed using the χ^2^ test or Fisher’s exact test, where appropriate. Confidence intervals (95% CI) were calculated using standard methods for mean differences or proportions, as appropriate. Clinically meaningful outcomes, such as ≥50% pain reduction or opioid discontinuation, were further analyzed using categorical responder rates and number needed to treat (NNT). A multivariable logistic regression was used to examine whether baseline characteristics (age, sex, BMI, duration of pain, baseline opioid use, and KL grade) predicted treatment response. Model fit and assumptions were verified using standard diagnostics. Statistical analyses were performed using GraphPad Prism 10.1.1 (GraphPad Software Inc., San Diego, CA, USA) and STATA 17.0 (StataCorp LLC, College Station, TX, USA). All *P* values were 2-tailed, and a *P* value of <.05 was considered statistically significant.

## Results

### Summary of participation

**Figure 1.**
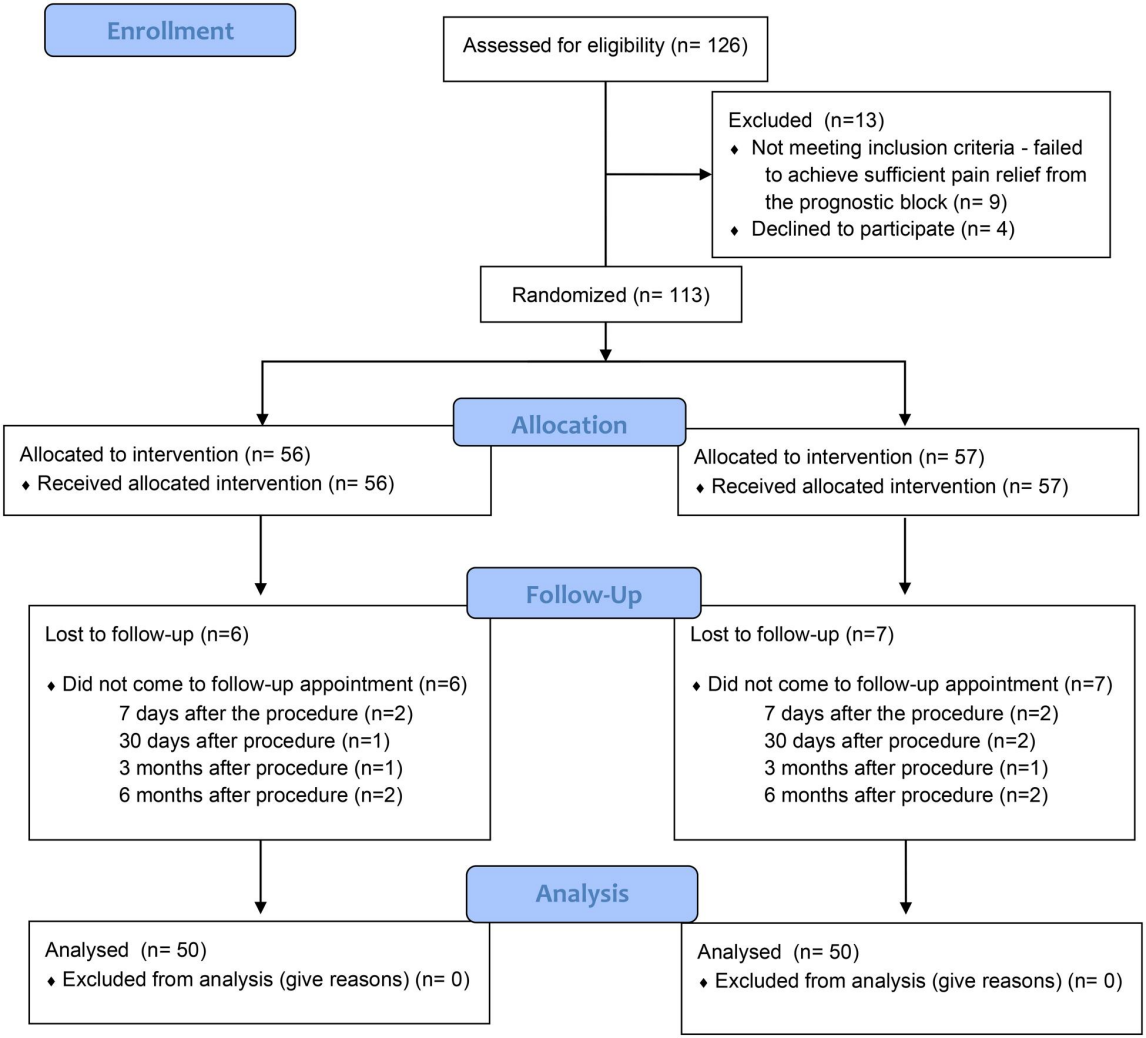
Consort flow diagram.

Of the 126 patients assessed for eligibility, 9 did not meet the inclusion criteria due to an insufficient response (<50% pain relief within 6 hours) to the prognostic genicular nerve block, and 4 refused to participate. The remaining 113 were randomly allocated to 2 groups. Thirteen patients were lost for follow-up due to not showing up for visits to the Pain Clinic. The remaining 100 patients were analyzed, as seen in Figure 1. No clinically relevant differences were apparent from group characteristics, as shown in Table 1.

**Table 1.** Baseline characteristics.

	Control group (n = 50)	Neurolysis (n = 50)	*P* value
Age	79.52 ± 8.45 (65-92)	79.74 ± 7.42 (66-92)	.9712
BMI	30.01 ± 2.50 (25.4-34.0)	29.00 ± 2.18 (24.4-36.4)	.0999
M/F	22/28	24/26	.8411
Duration of pain (months)	18.60 ± 5.71 (5-24)	17.88 ± 6.22 (6-29)	.6042
Morphine equivalent—daily dose (mg)	12.50 ± 4.20 (5-20)	12.58 ± 4.74 (5-20)	.9849
KL grade	3.58 ± 0.50 (3-4)	3.54 ± 0.50 (3-4)	.8405
3	23	25	
4	27	25	
NRS—before procedure	6.76 ± 0.89 (4-8)	6.48 ± 0.97 (5-8)	.1340
Neurological deficits	0	0	1.0000
Total opioid consumption (milligrams of oral morphine per day)	33.10 ± 20.31	31.60 ± 18.73	.7660
EQ-5D-5L Mobility	2.52 ± 0.89 (1-5)	2.48 ± 0.91 (1-4)	.8962
EQ-5D-5L Self-Care	1.44 ± 0.50 (1-2)	1.34 ± 0.52 (1-2)	.3030
EQ-5D-5L Usual Activity	2.32 ± 0.94 (1-4)	2.56 ± 0.73 (1-4)	.0664
EQ-5D-5L Pain/Discomfort	3.30 ± 0.84 (2-4)	3.30 ± 0.81 (2-4)	.9587
EQ-5D-5L Anxiety/Depression	1.48 ± 0.50 (1-2)	1.46 ± 0.50 (1-2)	.8451
EQ-5D-5L total	11.06 ± 1.36 (5-20)	11.14 ± 1.63 (5-20)	.9972

Data are expressed as a number or mean (SD). Values are mean (SD) or number.

Abbreviations: BMI, body mass index; DEM, dexmedetomidine; DEX, dexamethasone; F, female; K-L, Kellgren-Lawrence; M, male; NRS, numerical rating scale; SD, sstandard deviation.

### Primary outcomes

Patients in the neurolysis group demonstrated significantly lower NRS scores at all time points—7 days, 30 days, 3 months, and 6 months post-procedure—compared to the control group (all *P* < .0001), as detailed in Table 2. The mean differences in NRS scores ranged from −3.54 to −3.84, with 95% confidence intervals indicating consistent and clinically significant benefit.

**Table 2.** Primary outcomes and clinically meaningful pain reduction.

Time Point	Control group (n = 50)	Neurolysis group (n = 50)	*P* value	Mean NRS difference (95% CI)	≥50% Pain reduction—control (95% CI)	≥75% Pain reduction—control (95% CI)	100% Pain reduction—control (95% CI)	≥50% Pain reduction—neurolysis (95% CI)	≥75% Pain REDUCTION—neurolysis (95% CI)	100% Pain reduction—neurolysis (95% CI)	Absolute risk difference (95% CI)
**7 days**	6.70 ± 0.89	3.00 ± 0.78	<.0001	−3.70 (-4.14 to 3.26)	4/50 (8%, 2.2%-19.2%)	1/50 (2%, 0.05%-10.5%)	0/50 (0%, 0%-7.1%)	42/50 (84%, 70.9%-92.8%)	27/50 (54%, 39.3%-68.2%)	8/50 (16%, 7.2%-29.1%)	76% (60.6%-87.1%)
**30 days**	6.58 ± 0.78	2.74 ± 0.69	<.0001	−3.84 (-3.14 to -3.45)	5/50 (10%, 3.3%-21.8%)	2/50 (4%, 0.5%-13.7%)	1/50 (2%, 0.05%-10.5%)	43/50 (86%, 73.3%-94.2%)	26/50 (52%, 37.4%-66.3%)	7/50 (14%, 5.8%-26.7%)	76% (61.2%-87.3%)
**3 months**	6.44 ± 1.03	2.90 ± 0.76	<.0001	−3.54 (-4.02 to -3.06)	6/50 (12%, 4.5%-24.3%)	3/50 (6%, 1.3%-16.5%)	1/50 (2%, 0.05%-10.5%)	41/50 (82%, 68.6%-91.4%)	25/50 (50%, 35.8%-64.2%)	6/50 (12%, 4.5%-24.3%)	70% (53.6%-83.1%)
**6 months**	6.84 ± 1.00	3.10 ± 0.74	<.0001	−3.74 (-4.2 to 3.28)	5/50 (10%, 3.3%-21.8%)	2/50 (4%, 0.5%-13.7%)	1/50 (2%, 0.05%-10.5%)	40/50 (80%, 66.3%-89.9%)	23/50 (46%, 31.8%-60.7%)	9/50 (18%, 8.9%-31.4%)	70% (53.0%-82.4%)

Clinically meaningful reduction was defined as a ≥ 50% decrease in baseline NRS score. Values are presented as mean ± SD for continuous variables and n/N (% with 95% CI) for categorical outcomes. Worst-case imputation assumes all dropouts in the neurolysis group were non-responders and all dropouts in the sham group were responders. Statistical significance remained (*P* < .001).

At 6 months, a clinically meaningful reduction in pain, defined as a ≥50% decrease in baseline NRS score, was achieved by 80% of patients in the neurolysis group (95% CI, 66.3%-89.9%) versus only 10% in the control group (95% CI, 3.3%-21.8%), resulting in an absolute risk difference of 70% (95% CI, 53.0%-82.4%). Notably, a ≥ 75% reduction in pain was observed in 46% of patients receiving neurolysis (95% CI, 31.8%-60.7%) compared to 4% in the control group (95% CI, 0.5%-13.7%). Complete pain relief (100%) was achieved by 18% in the neurolysis group (95% CI, 8.9%-31.4%) versus 2% in the control group (95% CI, 0.05%-10.5%) (*P* = .008). A consistent pattern of response favoring neurolysis was observed across all follow-up periods, including at 7 days, 30 days, and 3 months, demonstrating both rapid onset and sustained efficacy.

These results demonstrate that ethanol neurolysis provides not only statistically significant but also clinically meaningful and sustained pain relief in patients with symptomatic knee osteoarthritis.

### Secondary outcomes

Patients in the neurolysis group demonstrated significantly lower mean daily opioid consumption, expressed in milligrams of oral morphine equivalent, at all follow-up time points compared to the sham control group, as seen in Table 3. Specifically, at 7 days post-procedure, the neurolysis group reported 5.3 ± 4.2 mg/day versus 14.1 ± 4.9 mg/day in the control group (mean difference: −8.8; 95% CI, −10.2 to −7.4; *P* < .0001). At 30 days, the values were 4.8 ± 3.9 mg/day versus 13.7 ± 5.1 mg/day (mean difference: −8.9; 95% CI, −10.4 to −7.4; *P* < .0001). At 3 months, opioid use remained reduced at 5.1 ± 4.4 mg/day versus 13.9 ± 5.2 mg/day (mean difference: −8.8; 95% CI, −10.3 to −7.3; *P* < .0001), and at 6 months, opioid use remained reduced at 5.2 ± 4.3 mg/day versus 13.8 ± 5.0 mg/day (mean difference: −8.6; 95% CI, −10.1 to −7.1; *P* < .0001).

**Table 3.** Mean daily opioid consumption (mg morphine equivalent ± SD).

Time Point	Neurolysis group (n = 50)	Control group (n = 50)	Mean difference (95% CI)	*P*-value	Number of opioid-free patients (neurolysis group)	NNT for opioid cessation
**7 days**	5.3 ± 4.2	14.1 ± 4.9	−8.8 (−10.2 to −7.4)	<.0001	35/50 (70%)	1.4
**30 days**	4.8 ± 3.9	13.7 ± 5.1	−8.9 (−10.4 to −7.4)	<.0001	37/50 (74%)	1.3
**3 months**	5.1 ± 4.4	13.9 ± 5.2	−8.8 (−10.3 to −7.3)	<.0001	37/50 (74%)	1.3
**6 months**	5.2 ± 4.3	13.8 ± 5.0	−8.6 (−10.1 to −7.1)	<.0001	35/50 (70%)	1.4

Proportion of patients opioid-free at each time point was lower in the sham group (0%) compared to the neurolysis group (70%-74%). Worst-case imputation analysis confirmed significant group differences.

Additionally, a higher proportion of patients in the neurolysis group were opioid-free at each time point (70% at 7 days and 6 months, 74% at 30 days and 3 months). In contrast, all patients in the control group continued to require opioids. The NNT to achieve opioid cessation ranged between 1.3 and 1.4 across follow-ups, highlighting the clinical impact of genicular neurolysis in reducing opioid dependence.

We did not observe neurological deficits in both groups at all time points (7 days, 30 days, 3 months, and 6 months after the procedure).

The total EQ-5D-5L health score was significantly lower in the neurolysis group compared to the sham group at all follow-up intervals: 7 days (6.90 ± 0.79 vs. 10.84 ± 1.63; mean difference: 3.94, 95% CI, 3.44 to 4.44; *P* < .0001), 30 days (6.88 ± 0.77 vs. 10.88 ± 1.70; mean difference: 4.00, 95% CI, 3.48 to 4.52; *P* < .0001), 3 months (7.04 ± 0.83 vs. 10.98 ± 1.68; mean difference: 3.94, 95% CI, 3.42 to 4.46; *P* < .0001), and 6 months post-procedure (7.82 ± 1.04 vs. 11.30 ± 1.69; mean difference: 3.48, 95% CI, 2.93 to 4.03; *P* < .0001), as seen in Table 4.

**Table 4.** Health Questionnaire—EQ-5D-5L.

		Control group (n = 50)	Neurolysis (n = 50)	*P*-value	Mean difference (95% Cl)
Mobility	7 days after procedure	2.34 (0.77)	1.20 (0.40)	<.0001	1.14 (0.90 to 1.38)
	30 days after procedure	2.34 (0.80)	1.14 (0.35)	<.0001	1.20 (0.96 to 1.44)
	3 months after procedure	2.30 (0.76)	1.12 (0.33)	<.0001	1.18 (0.95 to 1.41)
	6 months after procedure	2.38 (0.75)	1.36 (0.48)	<.0001	1.02 (0.77 to 1.27)
Self-care	7 days after procedure	1.52 (0.50)	1.08 (0.27)	<.0001	0.44 (0.28 to 0.60)
	30 days after procedure	1.54 (0.50)	1.08 (0.27)	<.0001	0.46 (0.30 to 0.62)
	3 months after procedure	1.62 (0.49)	1.16 (0.37)	<.0001	0.46 (0.29 to 0.63)
	6 months after procedure	1.74 (0.44)	1.22 (0.42)	<.0001	0.52 (0.35 to 0.69)
Dual Activities	7 days after procedure	2.16 (0.89)	1.60 (0.49)	.0003	0.56 (0.28 to 0.84)
	30 days after procedure	2.24 (0.92)	1.60 (0.49)	<.0001	0.64 (0.35 to 0.93)
	3 months after procedure	2.26 (0.92)	1.64 (0.48)	<.0001	0.62 (0.33 to 0.91)
	6 months after procedure	2.20 (0.90)	1.64 (0.48)	.0003	0.56 (0.28 to 0.84)
Pain/Discomfort	7 days after procedure	3.30 (0.84)	1.64 (0.49)	<.0001	1.66 (1.39 to 1.93)
	30 days after procedure	3.30 (0.82)	1.66 (0.48)	<.0001	1.64 (1.38 to 1.90)
	3 months after procedure	3.32 (0.82)	1.72 (0.45)	<.0001	1.60 (1.34 to 1.86)
	6 months after procedure	3.32 (0.82)	2.16 (0.79)	<.0001	1.16 (0.84 to 1.48)
Anxiety/Depression	7 days after procedure	1.40 (0.49)	1.48 (0.50)	.5459	−0.08 (-0.27 to 0.11)
	30 days after procedure	1.40 (0.49)	1.48 (0.50)	.5459	−0.08 (-0.27 to 0.11)
	3 months after procedure	1.40 (0.49)	1.48 (0.50)	.5459	−0.08 (-0.27 to 0.11)
	6 months after procedure	1.44 (0.50)	1.66 (0.59)	.7200	−0.22 (-0.43 to -0.01)
**EQ-5D-5L (Total score)**	**7 days after procedure**	10.84 (1.63)	6.90 (0.79)	<.0001	3.94 (3.44 to 4.44)
	**30 days after procedure**	10.88 (1.70)	6.88 (0.77)	<.0001	4.00 (3.48 to 4.52)
	**3 months after procedure**	10.98 (1.68)	7.04 (0.83)	<.0001	3.94 (3.42 to 4.46)
	**6 months after procedure**	11.30 (1.69)	7.82 (1.04)	<.0001	3.48 (2.93 to 4.03)

Values are mean (SD) or number.

Additionally, the proportion of patients achieving the minimal clinically important difference (MCID) in EQ-5D-5L score was significantly higher in the neurolysis group than in the sham group at all time points. At 7 days, 76% (95% CI, 62%-87%) of neurolysis patients met the MCID, compared to 12% (95% CI, 5%-25%) in the sham group (*P* < .001). This difference persisted at 30 days (80% vs. 14%), 3 months (82% vs. 16%), and 6 months (74% vs. 20%), all *P* < .001, indicating consistent, clinically meaningful improvements in quality of life (Table 5).

**Table 5.** EQ-5D-5L outcomes with MCID analysis.

Time point	% Patients meeting MCID (neurolysis)	95% CI	% Patients meeting MCID (sham)	95% CI	*P* value
**7 days**	76%	62%-87%	12%	5%-25%	<.001
**30 days**	80%	66%-90%	14%	6%-27%	<.001
**3 months**	82%	69%-92%	16%	7%-30%	<.001
**6 months**	74%	60%-85%	20%	10%-35%	<.001

Even under worst-case assumptions, the proportion of patients achieving MCID remained significantly higher in the neurolysis group (*P* < .01).

### Worst-case sensitivity analysis

To assess the robustness of our findings, we performed a worst-case imputation analysis consistent with CONSORT guidelines. In this analysis, all patients lost to follow-up in the neurolysis group (n = 7) were considered treatment failures (non-responders), and those lost in the sham group (n = 6) were assumed to have achieved clinically meaningful success. Despite this conservative assumption, statistically significant differences in primary outcomes (≥50% NRS reduction) and opioid cessation rates between the neurolysis and sham groups persisted (*P* < .001). This analysis reinforces the robustness of the observed treatment effects and highlights the clinical utility of genicular nerve chemical neurolysis.

### Subgroup and multivariable analysis

A subgroup and multivariable regression analysis was performed to evaluate whether baseline clinical and demographic variables influenced treatment response. Independent variables included age, sex, BMI, duration of knee pain, baseline opioid use, and KL grade. The dependent outcomes were (1) achievement of ≥50% pain relief on the NRS, (2) opioid cessation at each follow-up point, and (3) achieving the minimal clinically significant difference (MCID) on the EQ-5D-5L.

The regression model demonstrated that KL grade 4 was significantly associated with a reduced likelihood of achieving ≥50% pain relief (OR 0.62; 95% CI, 0.42-0.91; *P *= .018), compared to KL grade 3. However, age, sex, BMI, and duration of pain were not independently associated with treatment response. Baseline opioid use was not a significant predictor of opioid cessation (*P *> .05).

Subgroup analyses showed that opioid-free status at all follow-up points was consistently higher in the neurolysis group (70%-74%) compared to 0% in the sham group, yielding an NNT of 1.3-1.4 for opioid cessation. Similarly, significantly more patients in the neurolysis group achieved the EQ-5D-5L MCID at all follow-ups (74%-82%) versus 12%-20% in the sham group, even under worst-case scenario assumptions, confirming the robustness of these findings (*P *< .001), as seen in Table 6.

**Table 6.** Subgroup and multivariable regression analysis results.

Variable	Outcome	Odds ratio (95% CI)	*P* value
**KL grade 4 vs 3**	≥50% pain relief (NRS)	0.62 (0.42-0.91)	.018
**Age, sex, BMI, duration of pain**	≥50% pain relief (NRS)	NS	>.05
**Baseline opioid use**	Opioid cessation	NS	>.05

## Discussion

The present study confirms the efficacy of ultrasound-guided chemical ablation of genicular nerves with 95% ethanol in patients with symptomatic knee OA, demonstrating significant improvements in pain relief and quality of life. The study's results align with previous findings on neurolytic techniques for pain management in osteoarthritis,^19^ adding further evidence that genicular nerve ablation can be an effective intervention for patients who have not responded adequately to conservative measures such as NSAIDs, paracetamol, and co-analgesics.

A key strength of this study is its randomized, double-blind, and sham-controlled design, which minimizes bias and allows for a robust comparison between neurolysis and placebo. To our knowledge, this is one of the largest sham-controlled trials evaluating chemical neurolysis of genicular nerves to date, addressing a major gap in the literature where high-quality control data have been sparse. The sham-controlled design enhances internal validity and offers compelling support for the observed treatment effects.

The substantial reduction in pain scores (NRS) and opioid consumption in the neurolysis group, observed at all time points, underscores the superiority of this approach in managing refractory knee OA pain. Our findings are similar to studies regarding radiofrequency,^20–23^ cryoablation,^24^ and chemical ablation with phenol^12^^,^^25^ of the genicular nerves in treating knee OA pain. Compared to phenol, ethanol has demonstrated a comparable neurolytic efficacy but offers certain practical advantages. Unlike phenol, ethanol is widely available, inexpensive, and does not require refrigeration or special handling precautions.^26^^,^^27^ Additionally, ethanol's faster onset of neurolytic action and higher lipid solubility may allow for more predictable lesioning, especially under ultrasound guidance.^28^ About safety, both agents have low systemic toxicity when used in controlled volumes. Still, ethanol’s better visualization under real-time hydro-dissection and shorter tissue half-life may confer a marginally improved safety profile.^29^

Additionally, the consistent improvement in EQ-5D-5L quality of life scores in the neurolysis group suggests that the benefits of pain reduction extend beyond mere symptom control, positively affecting the broader well-being of patients. There is a lack of research in the existing literature regarding the impact of nerve ablation on the quality of life in patients with knee osteoarthritis. To our knowledge, this study is the first to comprehensively evaluate the effects of chemical ablation of the genicular nerves on patient-reported quality-of-life outcomes in this population. By incorporating validated measures such as the EQ-5D-5L,^18^^,^^30^ our investigation provides novel insights into this intervention's broader functional and psychosocial benefits beyond pain reduction.

Notably, the absence of neurological complications throughout the follow-up period is notable, as it demonstrates the safety profile of ethanol-based neurolysis when guided by ultrasound, similar to that of other ultrasound-guided neurolytic procedures.^14^ Given the precise targeting of genicular nerves, this technique mitigates the risk of unintended nerve damage, a concern often associated with other neurolytic procedures.^31^ The consistency of this safety outcome across all time points further strengthens the clinical applicability of this approach.

The implications of reduced opioid consumption in the neurolysis group are particularly relevant in light of the ongoing opioid crisis.^32^^,^^33^ Patients receiving neurolysis required fewer opioids to manage their pain, indicating a potential role for this intervention in reducing opioid dependence in this population.^34–36^ Given the risks associated with long-term opioid use, including addiction and side effects, the ability to reduce opioid requirements without compromising pain control is a significant clinical advantage.^37^

A notable consideration in procedural technique is the use of ultrasound versus fluoroscopic guidance. Ultrasound offers the advantage of real-time visualization of adjacent soft tissues, blood vessels, and needle placement, reducing the risk of intravascular injection. It is also radiation-free and more accessible in many settings.^38^ Conversely, fluoroscopy enables bony landmark identification with high precision and may be preferred in obese patients or where ultrasound imaging is limited. Digital subtraction angiography with fluoroscopy can further enhance safety in neurolytic ethanol procedures. Both modalities have merits, and operator experience may guide the choice.^39^

### Limitations

Several limitations of the study should be acknowledged. The single-center design may limit the generalizability of the findings to other clinical settings or populations. Additionally, while the study population included patients with symptomatic knee OA (KL grade 3 or 4), further studies are warranted to determine whether similar outcomes can be achieved in younger or less severe OA patients. Future research could also explore the long-term efficacy of chemical neurolysis beyond the 6-month follow-up period used in this study and investigate potential benefits in combination with other non-pharmacological interventions, such as physical therapy or exercise programs.

## Conclusion

This study provides compelling evidence that chemical ablation of genicular nerves with 95% ethanol is a safe, effective, and well-tolerated option for managing refractory knee osteoarthritis pain in patients with symptomatic knee OA. The observed reductions in pain, opioid consumption, and improvements in quality of life without neurological complications suggest that this technique may be a valuable addition to the therapeutic armamentarium for knee OA, particularly in patients who have not responded to conventional treatments. Further multicenter studies and long-term follow-up data are needed to validate these findings and explore broader applications of this intervention.
